# Effects of Dietary Fat Level of Concentrate Mix on Growth Performance, Rumen Characteristics, Digestibility, Blood Metabolites, and Methane Emission in Growing Hanwoo Steers

**DOI:** 10.3390/ani14010139

**Published:** 2023-12-31

**Authors:** Hyunjin Cho, Sinyong Jeong, Kyewon Kang, Mingyung Lee, Seoyoung Jeon, Hamin Kang, Hanbeen Kim, Jakyeom Seo, Joonpyo Oh, Seongwon Seo

**Affiliations:** 1 Division of Animal and Dairy Sciences, Chungnam National University, 99 Daehak-ro, Yuseong-gu, Daejeon 34134, Republic of Korea; chohyunjin0927@gmail.com (H.C.); syjeong89@gmail.com (S.J.); kangkyewon26@gmail.com (K.K.); mingyung1203@gmail.com (M.L.); seoyoung203@gmail.com (S.J.); gkals0339@gmail.com (H.K.); 2 Department of Animal Science, Life and Industry Convergence Research Institute, Pusan National University, Miryang 50463, Republic of Korea; khb3850@pusan.ac.kr (H.K.); jseo81@pusan.ac.kr (J.S.); 3 Cargill Animal Nutrition Korea, Seongnam 13630, Republic of Korea; davidoh@cargill.com

**Keywords:** dietary fat, concentrate mix, laser methane detector, methane emission, Hanwoo

## Abstract

**Simple Summary:**

This study investigated the effect of varying levels of dietary fat in concentrate mixes on growth performance, rumen characteristics, digestibility, blood metabolites, and methane emissions in growing Hanwoo steers. The experiment was conducted for 12 weeks by feeding three concentrate mixes with different levels of dietary fat (i.e., 48, 74, 99 g/kg dry matter [DM] of concentrate mix). Increasing fat levels in the concentrate mix significantly reduced the intake of concentrate mix (*p* < 0.01); however, there were no significant adverse effects on the growth rate, feed efficiency, and digestibility (*p* > 0.05). Increasing fat levels also affected the characteristics of rumen fluid, increasing the proportion of propionate (*p* < 0.01) and decreasing the proportion of butyrate and acetate to propionate in the total volatile fatty acids (*p* < 0.01). Cholesterol in blood serum increased significantly with increasing fat levels (*p* < 0.01). The highest fat level showed 18% lower methane concentration in exhaled gas from eructation than the lowest (basal) fat level (*p* < 0.05). In conclusion, increasing the fat level up to 100 g/kg DM in the concentrate mix reduced methane concentration in the exhaled gas from eructation without altering growth performance in growing Hanwoo steers.

**Abstract:**

This study investigated the effect of different dietary fat levels in concentrate mixes on the growth performance, rumen characteristics, digestibility, blood metabolites, and methane emissions in growing Hanwoo steers. Thirty steers (386 ± 24.6 kg of body weight [BW]; 12 months old), blocked by BW, were randomly assigned to three dietary treatments with varying fat concentrations in concentrate mix (48, 74, and 99 g of ether extract per kg dry matte [DM]). The fat intake of the low-fat treatment represented 4.15% of the total dry matter intake (DMI), while the medium- and high-fat treatments accounted for 5.77% and 7.23% of total DMI, respectively. Concentrate mix DMI decreased with increasing fat level (*p* < 0.01). The growth rate and digestibility did not significantly differ based on the fat level (*p* > 0.05). As the fat level increased, propionate in the total ruminal volatile fatty acids increased, and butyrate and acetate-to-propionate decreased (*p* < 0.01). Cholesterol in blood serum increased significantly with increasing dietary fat levels (*p* < 0.01). Methane emissions exhibited a linear decrease with increasing fat level (*p* < 0.05). In conclusion, elevating fat content in the concentrates up to 100 g/kg DM reduced methane emissions without compromising the growth performance of growing Hanwoo steers.

## 1. Introduction

Efforts are being made globally to reduce greenhouse gas (GHG) emissions, adhering to the Paris Agreement, which seeks to restrict the rise in global average temperatures to below 2 °C above pre-industrial levels, with an ambitious goal of limiting it to 1.5 °C [1]. Mitigating methane (CH_4_) emissions, which have a short lifespan in the atmosphere but a substantial global warming potential, can immediately reduce GHG [2]. Because CH_4_ emissions from ruminants account for 16% of the total global CH_4_ emissions [3] and cause energy loss in ruminants [4], reducing them is an ongoing research theme. To this end, various strategies and technologies are being researched and developed to reduce CH_4_ emissions by ruminant animals.

No livestock producer would favor strategies that negatively affect productivity, even if they effectively reduce CH_4_ emissions of ruminants. A dietary strategy that maintains or improves productivity and reduces CH_4_ emissions can be an approach to alleviate these concerns [5]. In particular, adding dietary fat can be a promising strategy to reduce CH_4_ emissions without negatively affecting productivity. It can lower CH_4_ emissions from enteric fermentation in ruminants through several mechanisms, including inhibitory effects on methanogens and protozoa, hydrogenation of unsaturated fatty acids (UFAs), promotion of propionate production, and limiting enteric fermentation [2,6,7,8]. Additionally, given its higher energy density compared to other nutrients such as carbohydrates and protein, fat can positively influence animal productivity [9].

Numerous meta-analyses and reviews have reported that dietary fat reduces the CH_4_ emissions of ruminants. Grainger and Beauchemin [10] conducted a meta-analysis on the effect of dietary fat on CH_4_ yield in beef, dairy, and sheep and demonstrated a statistically significant negative relationship between dietary fat level and CH_4_ yield, without any discernible effects based on animal species, fatty acid type, or fat source. In contrast, Patra [11] reported that fatty acid composition affects CH_4_ reduction differently, with fatty acids C12:0 and C18:3 having the greatest CH_4_ suppression effect. However, Patra [11] cautioned that a dietary fat content exceeding 6% of the dry matter (DM) not only reduces CH_4_ emissions but also hinders digestion and rumen fermentation, potentially lowering milk yield, and therefore necessitates careful consideration. A recent meta-analysis by Arndt et al. [12] also showed that fat supplementation diminished dry matter intake (DMI) and fiber digestibility (by −6 and −4%, respectively), concurrently reducing total CH_4_ production and yield (by −19 and −15%, respectively) without adversely affecting average daily gain (ADG).

Nevertheless, the effect of dietary fat supplementation on reducing CH_4_ emissions may vary depending on the cattle’s physiological stage and species. Additionally, since a high fat content in diets can lead to a reduced growth rate by reducing DMI and digestibility [11,13], an evaluation of the appropriate dietary fat level to reduce CH_4_ emissions without adversely affecting the growth rate is needed. To the best of our knowledge, no study has evaluated the effect of dietary fat levels on CH_4_ emissions in growing Hanwoo steers. Therefore, we assessed the impact of varying levels of dietary fat in concentrate mixes on the growth performance, rumen characteristics, digestibility, blood metabolites, and CH_4_ emissions in growing Hanwoo steers.

## 2. Materials and Methods

This study was conducted at the Center for Animal Science Research, Chungnam National University, Republic of Korea. The use of animals and the protocols for this experiment were reviewed and pre-approved by the Chungnam National University Animal Research Ethics Committee (202109A-CNU-108).

### 2.1. Animals, Housing, and Diets

Thirty 12-month-old growing Hanwoo steers (386 ± 24.6 kg), blocked by initial body weight (BW) and estimated breeding values for carcass BW, were randomly assigned to one of three dietary treatments based on completely randomized block design [14]. Two steers of similar BW were grouped within a block and housed together in a pen (5 m × 5 m) equipped with a forage feed bunk, which allowed us to measure individual feed intake automatically by identifying each animal using a radio-frequency identification tag attached to them (Dawoon, Co., Incheon, Republic of Korea). The experiment lasted for three months, with a seven-day adaptation period beforehand.

The diets were provided twice daily at 08:00 and 18:00. Forage, Klien hay (*Panicum coloratum*), was available ad libitum. Two types of concentrate mix were prepared: one low-fat concentrate mix containing 48 g of ether extract (EE) and a high-fat concentrate mix containing 99 g of EE per kg DM. There were three dietary treatments: (1) low fat (100% low-fat concentrate mix), (2) medium fat (50% low-fat contrate mix and 50% high-fat concentrate mix), and (3) high fat (100% high-fat concentrate mix). The diets were formulated to target an ADG of 0.9 kg/d, meeting the nutritional requirements following the Korean feeding standards for growing Hanwoo steers [15]. The formulation and chemical compositions of the experimental diets are described in Table 1 and Table 2.

### 2.2. Measurement and Sample Collection

Daily forage intake was recorded individually through the forage feed bunk (Dawoon, Co., Incheon, Republic of Korea), and concentrate mix daily intake was recorded individually by measuring the feed offered and refused. Every four weeks, each steer’s recorded daily feed intakes were processed. Intakes with more or less three times the SD from the mean intake were treated as outliers and removed. The intakes of the day when management activities took place that could have affected feed intakes, such as bedding replacement, BW measurement, and sampling periods, were also removed. The BW of steers was measured every four weeks before morning feeding, and the feed samples for chemical composition analysis were sampled once every four weeks.

After 12 weeks, fecal samples were spot-sampled eight times over the course of four consecutive days at nine h intervals (d 1, 17:00; d 2, 02:00, 11:00, 20:00; d 3, 05:00, 14:00, 23:00; and d 4, 08:00) for a total of 15 steers (5 per treatment). Fecal samples collected were dried at 65 °C for 72 h, and the dried fecal samples at each time point were pooled on an equal weight basis by steers.

Rumen fluid was collected three times (−1, +3, and +6 h after morning feeding) on three consecutive days after 5 and 11 weeks for all steers using an oral stomach tube, as described by Lee et al. [16]. Briefly, the initially collected rumen fluid was discarded (approximately 300 mL), and 400 mL of rumen fluid was subsequently collected in a glass flask. After collection, the pH of the rumen fluid was immediately measured, and 10 mL each was subsampled for ammonia (NH_3_-N) and volatile fatty acid (VFA) analysis and stored at −20 °C until analysis.

After 12 weeks, approximately 10 mL of blood was collected from the jugular vein of all steers before morning feeding. The collected blood was placed into a serum separator tube (BD Vacutainer; BD and Co., Franklin Lakes, NJ, USA). Serum was obtained by centrifugation at 1300× *g* for 15 min at 4 °C and frozen at −80 °C until analysis.

Methane emissions of all steers were measured four times daily (−2, −1, +1, and +2 h after morning feeding) using a laser methane detector (LMD; Laser Methane Mini, Tokyo Gas Engineering Solutions Co., Ltd., Tokyo, Japan) for five consecutive days after 2, 6, and 10 weeks, as described by Kang et al. [17]. Briefly, with the LMD installed stably on a tripod, the visible laser was aimed at the steer’s nose from a distance of 1 m, and CH_4_ emissions were measured every 0.5 s for 6 min. The measurements were duplicated.

### 2.3. Chemical Analyses and Data Processing

Chemical analyses were performed as described by Jeon et al. [18]. The feed and fecal samples were dried at 60 °C for 96 h and ground through a cyclone mill (Foss, Hillerød, Denmark) fitted with a 1 mm screen. The nutrient composition of the feed samples was analyzed at Cumberland Valley Analytical Services Inc. (Hagerstown, MD, USA). The content of DM (#934.15), crude protein (#990.03), EE (#920.39), acid detergent fiber (#973.18), and ash (#942.05) were determined. Crude protein was calculated as 6.25 times the nitrogen content, and the total nitrogen was measured using the Dumas method using a Leco FP-528 Nitrogen Combustion Analyzer (Leco Inc., Saint Joseph, MI, USA). The acid detergent lignin (ADL) content was analyzed and the neutral detergent fiber (aNDF) content was analyzed using a heat stable amylase and expressed inclusive of residual ash. The soluble protein, neutral detergent insoluble crude protein (NDICP) and acid detergent insoluble crude protein (ADICP) contents were also determined. The contents of ethanol soluble carbohydrate (ESC), starch, and macro- and micro-mineral content were determined. The NH_3_-N concentration of the rumen fluid was determined as follows. Following re-centrifugation of the rumen fluid at 21,000× *g* for 15 min, 20 μL of the supernatant was mixed with 1 mL of phenol color reagent and 1 mL of alkali hypochlorite reagent. The mixture was then incubated in a water bath for 15 min at 37 °C. After being mixed with 8 mL of distilled water, the optical density of the mixture was measured at 630 nm using a spectrophotometer (UV-1800, Shimadzu, Kyoto, Japan). In order to determine the VFA concentration of rumen fluid, rumen fluid supernatant (1 mL) was mixed with 0.2 mL of metaphosphoric acid (250 g/L) and kept at 4 °C for 30 min. Following centrifugation of the mixture at 21,000× *g* for 10 min at 20 °C, the supernatant was injected into a gas chromatograph (HP 6890, Hewlett-Packard Co., Palo Alto, CA, USA) equipped with a flame ionization detector and capillary column (Nukol Fused silica capillary column 30 m × 0.25 mm × 0.2 μm, Supelco Inc., Bellefonte, PA, USA). The temperature of the oven, injector, and detector was 90 °C to 180 °C, 185 °C, and 210 °C, respectively. Nitrogen was used as the carrier gas at a flow rate of 40 mL/min. The serum was analyzed for total protein, aspartate transaminase, alanine transaminase, glucose, total cholesterol, triglycerides, non-esterified fatty acid, blood urea nitrogen, creatinine, calcium, inorganic phosphate, magnesium, and albumin using kits purchased from Wako Pure Chemical Industries, Ltd. (Osaka, Japan) and a clinical auto-analyzer (Toshiba Accute Biochemical Analyzer-TBA-40FR, Toshiba Medical Instruments, Tokyo, Japan). Additionally, nutrient digestibilities were determined using feed internal markers, which were analyzed for acid-insoluble ash (AIA) in the feed and feces, as described in Van Soest et al. [19].

Methane emission data were separated into respiration and eructation by detecting CH_4_ concentration peaks using the automatic multi-scale peak detection package in R software [20]; version 4.0.2, R Foundation for Statistical Computing, Vienna, Austria and fitting a double normal distribution using the mixdist package in R software. The mean of a normal distribution represents the mean CH_4_ concentration per day as the mean of four time of day values, assuming representative CH_4_ concentrations of exhaled gas from the pathway during that time period [17].

### 2.4. Statistical Analysis

All data were analyzed using the PROC MIXED procedure of SAS (SAS Institute Inc., Cary, NC, USA). The blocks (i.e., initial BW and breeding value for carcass weight) were treated as random effects.

*y*_ij_ = *μ* + *τ*_i_ + *e*_ij_

where *y*_ij_ is the jth observation (j = 1–30) in the ith treatment (i = 1–3), *μ* is the overall mean, *τ*_i_ is the fixed effect of the ith treatment, and *e*_ij_ is the unexplained random effect on the jth observation in the ith treatment.

The normality of each parameter was assessed using the Shapiro–Wilk test of SAS (SAS Institute Inc., Cary, NC, USA), and it was determined that all parameters exhibited normality. Individual means were also compared using Tukey’s test. To test the effects of the different treatments on the rumen parameters and CH_4_ emissions, the data were analyzed as repeated measures to account for the correlation between repeated measurements of each animal. For this analysis, no structure was assumed for the variance–covariance matrix. Significance was declared at *p* < 0.05, and a trend was discussed at 0.05 ≤ *p* < 0.1.

## 3. Results

There was no significant difference in ADG among the treatments (*p* > 0.05; Table 3). No statistical differences were also observed for forage and total DMI and feed efficiency among the treatments (*p* > 0.05). However, concentrate DMI decreased significantly as the level of dietary fat increased. The concentrate DMI of the high-fat treatment was 0.4 and 0.5 kg/d lower than the medium-fat and low-fat treatments, respectively (*p* = 0.001). As per the design of this study, the total EE intake increased significantly with an increase in dietary fat levels (*p* < 0.001). The total EE intake in the high-fat diet, with a value of 0.64 kg/d, was significantly higher (*p* < 0.001) than the medium- (0.53 kg/d) and low-fat (0.39 kg/d) diets.

No effect of dietary fat levels on the digestibility of nutrients was observed (*p* > 0.05; Table 4). However, fiber digestibility tended to decrease linearly as the dietary fat level increased. Digestibility of neutral detergent fiber (NDF) and acid detergent fiber (ADF) tended to be the lowest in the high-fat treatment (52.25%, *p* = 0.089, and 31.24%, *p* = 0.074, respectively).

Dietary fat levels had no significant effect on rumen pH and ammonia (*p* > 0.05; Table 5). Likewise, there was no significant difference in total VFA among the treatments (*p* > 0.05), although there was a significant difference among the treatments in the proportion of certain VFA. The proportion of propionate decreased significantly with decreasing dietary fat levels (*p* < 0.001) and was lower in the low-fat treatment (191.8 mM) than in the medium- (203.0 mM) and high-fat treatments (212.6 mM).

Due to these differences, there was also a significant difference in the acetate-to-propionate (A:P) ratio among the treatments (*p* = 0.006). There was no significant difference in acetate among the treatments (*p* > 0.05), but the A:P ratio in the low-fat treatment (3.33 mM) was significantly higher than that in the medium- (3.09 mM) and high-fat treatments (3.02 mM). Contrary to the proportion of propionate, the proportion of butyrate decreased significantly with increasing dietary fat levels (*p* = 0.001) and was significantly lower in the high-fat treatment (112.1 mM) than in the medium- (127.7 mM) and low-fat treatments (125.7 mM).

No significant differences were observed for most blood metabolites (Table 6). However, cholesterol increased as the level of dietary fat increased (*p* = 0.001). Cholesterol was significantly higher in the high-fat treatment than in the low-fat treatment (140.1 vs. 103.6 mg/dL, respectively; *p* < 0.05).

The CH_4_ concentration measured in the respiratory exhaled gas did not display any significant alterations in response to variations in dietary fat (*p* > 0.05; Table 7). This observation remained consistent when CH_4_ concentrations in the exhaled respiratory gas were normalized to parameters such as DMI, NDF intake, forage NDF intake, and ADG (ppm/kg), with no noticeable differences between the treatment groups. Conversely, a significant impact of dietary fat on the CH_4_ concentrations in the gas exhaled during eructation was observed (*p* < 0.05). Specifically, an incremental rise in dietary fat was correlated with a decline in CH_4_ concentrations during eructation. Notably, the high-fat diet exhibited an 18% (11.6 ppm) reduction in CH_4_ concentrations compared to its low-fat counterpart (*p* = 0.017). Nevertheless, similar to the CH_4_ concentrations in the exhaled gas from respiration, there was no significant difference when CH_4_ concentrations were expressed as per DMI, NDF intake, forage NDF intake, and ADG (ppm/kg).

## 4. Discussion

Reducing CH_4_ emissions from ruminants is of significant interest, along with global efforts to reduce GHG emissions. Dietary fat supplementation has been evaluated as a strategy that can decrease CH_4_ emissions from ruminants without negatively affecting their productivity [9]. However, excessive levels of dietary fat can have adverse effects on production [11], making it necessary to evaluate the optimal fat level to prevent such negative impacts. The optimal dietary fat level may vary depending on the physiological stage or breed of the animal. Previous studies have not investigated the effect of dietary fat supplementation on CH_4_ emissions in growing Hanwoo steers, so this study aimed to clarify this issue.

It is well known that a high-fat diet decreases DMI and feed digestibility, negatively impacting animal performance [11]. To avoid the adverse effects of a high concentration of dietary fat, it is recommended that the fat content in the total diet does not exceed 7% DM [21]. Based on a meta-analysis, Patra [11] indicated that when the total dietary fat content exceeded 4.2% DM, the DMI decreased. Furthermore, when the fat content exceeded 6% DM, digestibility and rumen fermentation were altered, consistent with the results of this study. We also found that the total EE intake of the low-fat treatment was 4.15% DM of the total DMI, whereas the total EE intake of the medium- and high-fat treatments was 5.77% DM and 7.23% DM of the total DMI, respectively. Although there were no significant differences in forage and total DMI between the treatments (*p* > 0.05), the concentrate mix DMI decreased significantly with increasing dietary fat (*p* = 0.001). However, our study indicated that a reduction in concentrate DMI by increasing dietary fat levels in the concentrate mix did not adversely affect ADG. Additionally, there was a tendency for decreased fiber digestibility (i.e., NDF and ADF) as dietary fat levels increased, but there was no significant difference in nutrient digestibility. These results could be attributed to the feed provided to the steers, which mainly consisted of a concentrate mix. Beauchemin et al. [22] suggested that increasing the fat content in a diet based on a concentrated mix (i.e., low-fiber diets) could be more desirable, as it reduces the negative effects of fat. Therefore, an increase in dietary fat content in such a diet may be more advantageous. Additionally, recent studies have shown that decreased DMI and feed digestibility resulting from fat supplementation do not necessarily lead to reduced growth rates. A recent meta-analysis conducted by Arndt et al. [12] indicated that fat supplementation through oil and fat sources reduced DMI (−6%) and digestibility (−4%), but it did not affect milk yield and ADG. On the other hand, fat supplementation through oilseeds did not affect DMI and milk yield, but it decreased digestibility (−8%) and ADG (−13%).

Generally, starch in the concentrate mix increases the production of propionate [23]. However, in this study, the concentrate mix intake decreased significantly with increasing dietary fat levels, but the proportion of propionate increased, and the proportion of butyrate decreased. Previous studies indicated similar results. Onetti et al. [24] reported that supplementing animal fats to corn silage increased the proportion of propionate in Holstein cows. These findings were consistent with a meta-analysis and review paper by Patra [11] and Sun et al. [25], which reported that increasing dietary fat levels were associated with increased propionate and decreased butyrate and the A:P ratio. Unsaturated fatty acids in the fat content of the feed decrease the populations of methanogens and protozoa in the rumen [26]. As a result of these effects, the hydrogen (H_2_) within the rumen increases, causing excessive amounts of reduced NADH to be channeled into propionate production [27]. Furthermore, fats in diets can inhibit the production of butyrate by suppressing microorganisms such as *Butyrivibrio fibrisolvens* in the rumen [28,29].

In this study, we observed significant increases in cholesterol levels in the blood serum as the dietary fat level of the concentrate mix increased (*p* = 0.001). Several previous studies have reported results similar to those of this study. Bai et al. [30] reported that supplementing UFA to Angus bulls at a fat level of 5% DM to 7% DM had no impact on blood cholesterol levels. However, when saturated fatty acids were used to increase the fat level up to 7%, it increased blood cholesterol. Similarly, the supplement of rumen protected fat in the growing and early fattening stages of Hanwoo steers significantly increased blood cholesterol levels [31,32]. Elevated blood cholesterol was attributed to enhanced lipid absorption in the intestine and the increased presence of metabolites in the bloodstream, reflecting the dietary fat content [33]. The results from this study suggest that dietary fat supplementation was adequately implemented according to the experimental design.

Although the extent to which dietary fat reduces CH_4_ emissions may vary depending on factors such as the source of fat, fatty acid composition, and duration of fat supplementation, the reduction in CH_4_ emissions resulting from dietary fat supplementation has been demonstrated in numerous previous studies and well established through several meta-analyses and review papers. The extent of CH_4_ emissions reduction varies, decreasing by approximately 4% [11] to 5.6% [22] for each 1% increase in dietary fat supplementation. Several studies have determined the long-term effect of dietary fat in reducing CH_4_. In a meta-analysis conducted by Grainger and Beauchemin [10], which included six studies providing dietary fat supplementation from weeks 3 to 36, it was reported that a 1% increase in dietary fat resulted in a CH_4_ reduction ranging from 1.7% to 6.7%. A meta-analysis by Arndt et al. [12] indicated that the inclusion of oils or fats in the diet compared to oilseeds resulted in similar reductions in daily CH_4_ emissions (by 20% and 20%, respectively) and yield (by 15% and 14%, respectively). Consistent with earlier studies, we found that increasing dietary fat levels does not have a negative impact on productivity, while reducing CH_4_ emissions in growing Hanwoo steers. Increasing the fat content in the concentrate mix up to 100 g/kg DM resulted in a linear decrease in CH_4_ emissions from eructation, with a 5.85% reduction in CH_4_ emissions for every 1% increase in the concentrate’s fat content (R^2^ = 0.97, *p* = 0.005). These results align with the range of effects of fat supplementation on CH_4_ emissions, as indicated by previous meta-analyses, and suggest that dietary fat remains effective in reducing CH_4_ emissions even in diets based on concentrate mix.

Dietary fat supplements can lead to a reduction in CH_4_ emissions from ruminant enteric fermentation through various mechanisms, including the toxic effects on methanogens and protozoa, biohydrogenation of UFAs, increased propionate production, and reduced fiber digestibility by reducing bacteria [2]. However, in this study, hydrogenated fat was used to control the level of dietary fat, and since no effect of fat was observed on digestibility, it is considered that the significant effect might be due to the toxic effects of fat on methanogens and protozoa. Furthermore, as higher fat levels led to increased propionate production, it is likely that H_2_ in the rumen was primarily utilized for propionate production rather than for the biohydrogenation of UFAs. Sun et al. [25] have suggested that due to the limited availability of H_2_ for the biohydrogenation of UFAs in the rumen, the primary influence of dietary fat on CH_4_ emissions would likely be its direct impact on methanogens and rumen microbial population.

## 5. Conclusions

In this study, increasing the fat content in the concentrate mix up to 100 g/kg DM resulted in a linear decrease in CH_4_ emissions from eructation. Specifically, there was a 5.85% reduction in CH_4_ emissions for every 1% increment in the concentrate’s fat content. Notably, the high fat content did not adversely affect productivity, even though it decreased concentrate mix intake in growing Hanwoo steers.

## Figures and Tables

**Table 1:** Diet formulation of the experimental diets.

Ingredients (g/kg DM) ^1^	Concentrate Mix		
	Low Fat	Medium Fat	High Fat
Corn, ground	31	33	35
Wheat, ground	185	168	150
Rice bran	43	43	42
Hydrogenated fat	0	24	48
Corn gluten feed	302	277	252
Soybean hull	40	20	0
Molasses	55	55	55
DDGS	103	134	165
Palm kernel meal	166	166	165
CMS	17	17	17
Limestone	37	38	38
Salt	4	7	10
Vitamin and mineral mix ^2^	17	21	24

^1^ DM: dry matter, DDGS: distillers dried grains with soluble, CMS: condensed molasses soluble. ^2^ 33,330,000 IU/kg vitamin A, 40,000,000 IU/kg vitamin D, 20.86 IU/kg vitamin E, 20 mg/kg Cu, 90 mg/kg Mn, 100 mg/kg Zn, 250 mg/kg Fe, 0.4 mg/kg I, and 0.4 mg/kg Se.

**Table 2:** Analyzed chemical composition (g/kg DM or as stated) of the experimental diets.

Items ^1^	Concentrate Mix			Klein Hay
	Low Fat	Medium Fat	High Fat	
DM, g/kg as fed	898	900	901	906
OM	913	903	892	917
CP	184	186	187	115
SOLP	80	76	72	48
NDICP	35	34	33	35
ADICP	8	12	15	9
aNDF	356	353	350	685
ADF	153	164	174	373
ADL	30	33	35	46
Ether extract	48	74	99	30
Ash	87	98	108	83
Ca	16	17	17	4
P	8	8	7	2
K	12	11	10	27
Na	2	3	4	1
Cl	5	6	7	17
S	5	5	5	2
Mg	4	4	3	4
TDN	716	732	747	576
NEm, MJ/kg DM	7.6	7.8	8.0	5.3
NEg, MJ/kg DM	5.0	5.2	5.3	2.9
Total carbohydrates	681	644	606	772
NFC	358	323	288	122
Carbohydrate fraction, g/kg carbohydrate				
CA	84	101	117	83
CB1	357	328	299	16
CB2	85	72	59	58
CB3	363	375	386	700
CC	106	122	138	143
Protein fraction, g/kg CP				
PA + B1	435	410	385	414
PB2	377	409	441	283
PB3	145	118	91	224
PC	43	63	82	79

^1^ DM: dry matter, OM: organic matter, CP: crude protein, SOLP: soluble CP, NDICP: neutral detergent insoluble CP, ADICP: acid detergent insoluble CP, aNDF: neutral detergent fiber analyzed using a heat stable amylase and expressed inclusive of residual ash, ADF: acid detergent fiber, ADL: acid detergent lignin, TDN: total digestible nutrients, NEm: net energy for maintenance, NEg: net energy for growth, NFC: non-fiber carbohydrate, CA: carbohydrate A fraction; ethanol soluble carbohydrates, CB1: carbohydrate B1 fraction; starch, CB2: carbohydrate B2 fraction; soluble fiber, CB3: carbohydrate B3 fraction; available insoluble fiber, CC: carbohydrate C fraction; unavailable carbohydrate, PA + B1: protein A and B1 fractions; soluble CP, PB2: protein B2 fraction; intermediate degradable CP, PB3: protein B3 fraction; slowly degradable fiber-bound CP, PC: protein C fraction; unavailable CP.

**Table 3:** Effects of dietary fat levels of concentrate mixes (low-, medium-, and high-fat) on the growth performance of growing Hanwoo steers.

Items ^1^	Treatment			SEM	*p*-Value		
	Low Fat	Medium Fat	High Fat		Mean	Linear	Quadratic
Initial BW, kg	386	386	386	8.1	0.999	0.997	0.990
Final BW, kg	458	465	460	10.6	0.902	0.874	0.673
ADG, g/day	777.7	850.0	804.4	48.13	0.569	0.699	0.326
Intake, kg/day							
Concentrate DMI	5.9 ^a^	5.8 ^a^	5.4 ^b^	0.10	0.001	0.001	0.121
Forage DMI	3.4	3.4	3.4	0.18	0.990	0.983	0.890
Total DMI	9.3	9.2	8.8	0.20	0.161	0.074	0.510
Total EE	0.39 ^c^	0.53 ^b^	0.64 ^a^	0.010	<0.001	<0.001	0.116
FCR	12.5	11.2	11.1	0.66	0.258	0.142	0.457

^1^ BW: body weight, ADG: average daily gain, DMI: dry matter intake, EE: ether extract, FCR: feed conversion ratio. ^a–c^ Means that do not have common superscripts differ significantly within the treatments (*p* < 0.05).

**Table 4:** Effects of dietary fat levels of concentrate mixes on nutrient digestibility in growing Hanwoo steers.

Items ^1^	Treatment			SEM	*p*-value		
	Low Fat	Medium Fat	High Fat		Mean	Linear	Quadratic
DM, %	66.44	62.48	62.26	2.306	0.382	0.223	0.521
OM, %	68.48	64.98	64.46	2.266	0.421	0.234	0.603
CP, %	68.20	67.51	69.45	2.332	0.841	0.713	0.654
EE, %	74.61	70.42	73.47	4.254	0.776	0.853	0.501
aNDF, %	58.87	52.40	52.25	2.535	0.151	0.089	0.329
ADF, %	40.82	33.68	31.24	3.467	0.170	0.074	0.591

^1^ DM: dry matter, OM: organic matter, CP: crude protein, EE: ether extract, aNDF: neutral detergent fiber analyzed using a heat stable amylase and expressed inclusive of residual ash, ADF: acid detergent fiber.

**Table 5:** Effects of dietary fat levels of concentrate mixes on rumen characteristics in growing Hanwoo steers.

Items ^1^	Treatment			SEM	*p*-Value		
	Low Fat	Medium Fat	High Fat		Mean	Linear	Quadratic
pH	6.49	6.46	6.41	0.081	0.749	0.456	0.893
NH_3_-N, mg/dL	5.14	5.65	5.65	0.630	0.803	0.565	0.745
Total VFA, mM	64.8	67.8	64.6	2.60	0.622	0.951	0.332
Molar proportions, mmol/mol							
Acetate	632.5	622.8	632.2	5.21	0.329	0.961	0.137
Propionate	191.8 ^b^	203.0 ^a^	212.6 ^a^	3.19	<0.001	<0.001	0.842
Isobutyrate	14.3	13.2	13.2	1.16	0.743	0.509	0.694
Butyrate	125.7 ^a^	127.7 ^a^	112.1 ^b^	3.05	0.001	0.002	0.021
Isovalerate	18.9	16.4	15.8	1.31	0.222	0.102	0.561
Valerate	16.8	17.0	14.2	0.84	0.033	0.029	0.145
Acetate/Propionate	3.33 ^a^	3.09 ^b^	3.02 ^b^	0.07	0.006	0.002	0.320

^1^ VFA: volatile fatty acid. ^a,b^ Means that do not have common superscripts differ significantly within the treatments (*p* < 0.05).

**Table 6:** Effects of dietary fat levels of concentrate mixes on blood metabolites in growing Hanwoo steers.

Items ^1^	Treatment			SEM	*p*-Value		
	Low Fat	Medium Fat	High Fat		Mean	Linear	Quadratic
Total protein, g/dL	5.2	5.7	5.5	0.12	0.060	0.106	0.073
Urea, mg/dL	15.5	15.1	16.9	0.720	0.197	0.167	0.241
Glucose, mg/dL	56.2	63.9	59.6	2.25	0.072	0.295	0.040
NEFA, mEq/L	0.14	0.16	0.16	0.011	0.345	0.180	0.577
Albumin, mg/dL	3.1	3.2	3.1	0.08	0.922	0.964	0.692
Creatinine, mg/dL	1.0	1.1	1.1	0.03	0.059	0.019	0.795
Triglyceride, mg/dL	15.1	14.7	17.5	1.54	0.397	0.289	0.396
GOT, U/L	50.3	50.1	54.2	2.80	0.517	0.339	0.531
GPT, U/L	17.9	17.9	18.8	0.94	0.725	0.480	0.714
Cholesterol, mg/dL	103.6 ^b^	117.2 ^ab^	140.1 ^a^	7.26	0.005	0.001	0.605
Calcium, mg/dL	8.0	8.1	8.0	0.14	0.642	0.922	0.353
Phosphorus, mg/dL	7.0	7.0	7.1	0.18	0.892	0.636	0.974
Magnesium, mg/dL	2.0	2.1	2.0	0.04	0.128	0.141	0.156

^1^ NEFA: non-esterified fatty acid, GOT: glutamic oxaloacetic transaminase, GPT: glutamic pyruvic transaminase. ^a,b^ Means that do not have common superscripts significantly differ within the treatments (*p* < 0.05).

**Table 7:** Effects of dietary fat levels of concentrates mix on methane concentration of repeated measurement in growing Hanwoo steers.

Items ^1^	Treatment			SEM	*p*-Value		
	Low Fat	Medium Fat	High Fat		Mean	Linear	Quadratic
CH_4_ from respiration							
ppm	11.59	11.98	11.39	0.395	0.278	0.292	0.226
ppm/kg of DMI	1.24	1.29	1.29	0.040	0.623	0.806	0.351
ppm/kg of FDMI	3.37	3.48	3.39	0.153	0.323	0.806	0.141
ppm/kg of NDFI	2.58	2.66	2.66	0.086	0.586	0.918	0.308
ppm/kg of FNDFI	4.92	5.08	4.94	0.224	0.323	0.806	0.141
ppm/kg of ADG	18.78	16.12	15.11	1.944	0.858	0.718	0.680
CH_4_ from eructation							
ppm	63.82 ^a^	59.43 ^ab^	52.27 ^b^	2.728	0.017	0.005	0.992
ppm/kg of DMI	6.84	6.46	6.04	0.302	0.203	0.079	0.802
ppm/kg of FDMI	18.64	17.57	15.71	0.972	0.236	0.099	0.718
ppm/kg of NDFI	14.29	13.54	12.54	0.637	0.193	0.073	0.919
ppm/kg of FNDFI	27.21	25.65	22.93	1.419	0.236	0.099	0.718
ppm/kg of ADG	108.24	88.58	70.68	12.257	0.609	0.574	0.415

^1^ DMI: dry matter intake, FDMI: forage dry matter intake, NDFI: neutral detergent fiber intake, FNDFI: forage neutral detergent fiber intake, ADG: average daily gain. ^a,b^ Means that do not have common superscripts differ significantly within the treatments (*p* < 0.05).

## Data Availability

The data presented in this study are available on request from the corresponding author.
